# Supplementary material for: Live-cell mapping of organelle-associated RNAs via proximity biotinylation combined with protein-RNA crosslinking
Source: eLife. 2017 Dec 14;6:e29224. doi: 10.7554/eLife.29224 (PMC5730372; doi:10.7554/eLife.29224)
Supplement: Transparent reporting form [file elife-29224-transrepform.pdf]

eLIFE
1st Floor
24 Hills Road
Cambridge CB2 1JP, UK
P 01223 855340
W elifesciences.org
T @elife

eLife's transparent reporting form

We encourage authors to provide detailed information *within their submission* to facilitate the interpretation and replication of experiments. If you have any questions, please contact us: editorial@elifesciences.org.

Sample-size estimation

- You should state whether an appropriate sample size was computed when the study was being designed
- You should state the statistical method of sample size computation and any required assumptions
- If no explicit power analysis was used, you should describe how you decided what sample (replicate) size (number) to use

Please outline where this information can be found within the submission (e.g., page numbers or figure legends), or explain why this information doesn't apply to your submission:

Our research does not require sample-size estimation

Replicates

- You should report how often each experiment was performed
- You should include a definition of biological versus technical replication
- The data obtained should be provided and sufficient information should be provided to indicate the number of independent biological and/or technical replicates
- If you encountered any outliers, you should describe how these were handled
- Criteria for exclusion/inclusion of data should be clearly stated
- High-throughput sequence data should be uploaded before submission, with a private link for reviewers provided (these are available from both GEO and ArrayExpress)

Please outline where this information can be found within the submission (e.g., page numbers or figure legends), or explain why this information doesn't apply to your submission:

eLIFE
1st Floor
24 Hills Road
Cambridge CB2 1JP, UK
P 01223 855340
W elifesciences.org
T @elife

Figure 1B: page 33
Figure 1C: page 35
Figure 1D and Figure 1-figure supplement 1B,E-F: page 40
Figure 1-figure supplement 1A and 1C: page 38
Figure 1-figure supplement 1D: page 35
Figure 1-figure supplement 2: page 33
Figure 2A: page 33
Figure 2B-D and Figure 2-figure supplement 1A-F,H-K: page 40
Figure 2-figure supplement 1G: page 34
Figure 3C: page 33
Figure 3D: page 35
Figure 3E: page 38
Figure 3F and Figure 3-figure supplement 1B-F: page 40
Figure 3-figure supplement 1A: page 38

Statistical reporting

- Statistical analysis methods should be described and justified
- Raw data should be presented in figures whenever informative to do so (typically when N per group is less than 10)
- For each experiment, you should identify the statistical tests used, exact values of N, definitions of center, methods of multiple test correction, and dispersion and precision measures (e.g., mean, median, SD, SEM, confidence intervals; and, for the major substantive results, a measure of effect size (e.g., Pearson's r, Cohen's d)
- Report exact p-values wherever possible alongside the summary statistics and 95% confidence intervals. These should be reported for all key questions and not only when the p-value is less than 0.05.

Please outline where this information can be found within the submission (e.g., page numbers or figure legends), or explain why this information doesn't apply to your submission:

The calculation for the p-values on page 14 is explained on page 14
The calculation for the p-values in Figure 4D is explained in the figure legend of Figure 4
The calculation for the p-values in Figure 3E, Figure 1-figure supplement 1C, and Figure 3-figure supplement 1A is explained on page 14
The calculation for the p-values in RNA-Seq data in figure 1D, 2C-D, 3F, and Figure 2-figure supplement 1H is explained on page 44

(For large datasets, or papers with a very large number of statistical tests, you may upload a single table file with tests, Ns, etc., with reference to page numbers in the manuscript.)

eLife Sciences Publications, Ltd is a limited liability non-profit non-stock corporation incorporated in the State of Delaware, USA, with company number 5030732, and is registered in the UK with company number FC030576 and

Additional data files ("source data")

- We encourage you to upload relevant additional data files, such as numerical data that are represented as a graph in a figure, or as a summary table
- Where provided, these should be in the most useful format, and they can be uploaded as "Source data" files linked to a main figure or table
- Include model definition files including the full list of parameters used
- Include code used for data analysis (e.g., R, MatLab)
- Avoid stating that data files are "available upon request"

Please indicate the figures or tables for which source data files have been provided:

All the relevant data for plots have been provided in Supplementary files.
